# Editorial: Putting wild vegetables to work for sustainable agriculture and food security

**DOI:** 10.3389/fpls.2023.1268231

**Published:** 2023-09-28

**Authors:** Ganesh Chandrakant Nikalje, Vishnu D. Rajput, Georgia Ntatsi

**Affiliations:** ^1^ Department of Botany, Seva Sadan’s R. K. Talreja College of Arts, Science and Commerce, University of Mumbai, Ulhasnagar, India; ^2^ Academy of Biology and Biotechnology, Southern Federal University, Rostov-on-Don, Russia; ^3^ Laboratory of Vegetable Production, Department of Crop Science, Agricultural University of Athens, Athens, Greece

**Keywords:** agriculture, domestication, food security, hunger, wild vegetables

The global population is expected to reach 10 billion within the next 20 years. To feed this growing population, there is an urgent need to increase food production by more than 70% (Byerlee et al., 2008). The biggest problem is to provide healthy and balanced diet to growing population. However, in this era of climate change, due to infestation of different biotic and abiotic stresses the productivity of agricultural crops is decreasing day by day. The United Nation Development Programme (UNDP) on Sustainable Development Goals aims “to end poverty, protect the planet, and ensure that by 2030 all people enjoy peace and prosperity”. Extreme hunger and malnutrition remain a huge barrier to development in many countries. In 2022, about 9.2% of world population has faced hunger which was 7.9% in 2019. Nearly 2.4 billion global population has been suffered due to moderate or severe food insecurity (FAO, IFAD, UNICEF, WFP and WHO, 2023). According to UN estimates, there are 828 million people estimated to be chronically undernourished as of 2021, often as a direct consequence of environmental degradation, drought, and biodiversity loss (World Health Organization, 2022). This warrants a focus on different approaches to meet the challenges of hidden hunger as well as nutritional and food security. Among these approaches, the utilization of wild vegetables is a promising option.

Wild vegetables, also known as foraged vegetables or edible wild plants, are plants that grow in the wild and can be consumed by humans. These plants are not intentionally cultivated or planted by humans but rather occur naturally in their native habitats. They have been traditionally used for food, medicine, and other purposes by various cultures around the world. In addition, they are tolerant to adverse environmental conditions, and can be grown at a low cost (Duguma, 2020). However, the utilization of wild vegetables is restricted to rural areas. Rural communities are rich in these resources, but due to the lack of awareness and technological investment, wild vegetables remained underutilized. The domestication of wild vegetables and future research into them could contribute to establishing sustainable agriculture, advancing food security, and fostering economic development in rural areas (Leakey et al., 2022; Luo et al., 2022).

In this Research Topic, Liber et al. studied the domestication history of *Lentil* by combined genotyping by sequencing (GBS) of wild and domesticated accessions. They discovered about 87,647 SNPs, which confirmed presence of four groups and detected gene flow between cultivated *Lentil* varieties and *L. culinaris* subsp. *orientalis* (*Lentil* progenitor) at very low level. In addition, authors have identified two domesticated gene pools which are emerging in South West Asia, presence of admixed varieties confirming relaxed selection process. Only few numbers of alleles are involved in adaptation to environmental variables and domestication process.

To understand the mechanism of cucurbitacin biosynthesis, Zhao et al. carried out a comprehensive transcriptomics and metabolomic analysis of *Luffa acutangula* fruits from bitter and non-bitter genotypes. This comparative account revealed presence of bitterness specific metabolites such as isocucurbitacin B, cucurbitacin F, cucurbitacin D and 23, 24-dihydro cucurbitacin E in high concentration and high expression of genes involved in cucurbitacin biosynthesis pathway such as cytochrome P450s, *Bi* and acetyletransferase. In addition, abscisic acid and drought stress enhanced expression of these genes. This study may help in domestication of wild bitter cucurbits with high stress tolerance and less bitterness.

Takahashi et al. utilized stress tolerant wild *Vigna stipulacea* as potential wild plant for domestication purpose. Authors have utilized the isi2 mutant to identify the function of the gene VsPSAT1 in reducing seed hardness. This study highlights the potential of wild *Vigna* species to be ulitized as future crops because of their high stress tolerance and nutritive value. In literature, the utilization of edible wild halophytes as potential future crops has been demonstrated. For example, in previous studies, halophytes like *Chenopodium quinoa*, *Salicornia* sp. *Sesuvium portulacastrum* etc. were reported to have high abiotic stress tolerance, rich source of antioxidants, nutrients, therapeutic value and can be utilized as future crop (Nikalje et al., 2018).

In another study, Mashamaite et al. reviewed morphology, growth conditions, nutrition and utilization of wild *Cleome gynandra* plants. Authors have discussed the status of domestication of this plant in several African countries and concluded that *C. gynandra* can be a potential candidate to manage deficiency of micronutrients in post pandemic era.

In India, Mandal et al., studied the diversity of macrofungi associated with wild edible plants in Eastern India for food security of local tribals. Their study identified *Madhuca longifolia* as frequently cited plant and *Colocasia esculenta, Marsilea vestita* and *Termitomyces heimii* as culturally import plants. This study reveals that sustainable utilization of wild edibles can be a treasure trove for mitigating human hunger.

In total, specific strategies must be adopted to produce wild vegetables on a large scale in a sustainable manner. Agricultural techniques, including site selection, crop choice, optimization of cultivation methods, and regenerative practices, should incorporate the principles of sustainable agriculture and responsible foraging. Ultimately, we believe that research on the ‘Utilization of Wild Vegetables’ will underscore the necessity of targeting wild plants for domestication, potentially paving the way for future crops in the realm of sustainable agriculture and food security, particularly in the face of climate change.

## Author contributions

GCN: Conceptualization, Formal Analysis, Supervision, Writing – original draft, Writing – review & editing. GN: Writing – review & editing. VR: Writing – review & editing.
